# Intelligent diagnosis of major depression disease based on multi-layer brain network

**DOI:** 10.3389/fnins.2023.1126865

**Published:** 2023-03-16

**Authors:** Dan Long, Mengda Zhang, Jing Yu, Qi Zhu, Fengnong Chen, Fangyin Li

**Affiliations:** ^1^Zhejiang Cancer Hospital, Institute of Basic Medicine and Cancer (IBMC), Chinese Academy of Sciences, Hangzhou, Zhejiang, China; ^2^School of Automation, Hangzhou Dianzi University, Hangzhou, China; ^3^The College of Computer Science and Technology, Nanjing University of Aeronautics and Astronautics, Nanjing, China

**Keywords:** multi-layer brain function network, major depression disease (MDD), intelligent diagnosis, the pathological basis, deep learning

## Abstract

**Introduction:**

Resting-state brain network with physiological and pathological basis has always been the ideal data for intelligent diagnosis of major depression disease (MDD). Brain networks are divided into low-order networks and high-order networks. Most of the studies only use a single-level network to classify while ignoring that the brain works cooperatively with different levels of networks. This study hopes to find out whether varying levels of networks will provide complementary information in the process of intelligent diagnosis and what impact will be made on the final classification results by combining the characteristics of different networks.

**Methods:**

Our data are from the REST-meta-MDD project. After the screening, 1,160 subjects from ten sites were included in this study (597 MDD and 563 normal controls). For each subject, we constructed three different levels of networks according to the brain atlas: the traditional low-order network based on Pearson’s correlation (low-order functional connectivity, LOFC), the high-order network based on topographical profile similarity (topographical information-based high-order functional connectivity, tHOFC) and the associated network between them (aHOFC). Two sample *t*-test is used for feature selection, and then features from different sources are fused. Finally, the classifier is trained by a multi-layer perceptron or support vector machine. The performance of the classifier was evaluated using the leave-one-site cross-validation method.

**Results:**

The classification ability of LOFC is the highest among the three networks. The classification accuracy of the three networks combined is similar to the LOFC network. These are seven features chosen in all networks. In the aHOFC classification, six features were selected in each round but not seen in other classifications. In the tHOFC classification, five features were selected in each round but were unique. These new features have crucial pathological significance and are essential supplements to LOFC.

**Conclusion:**

A high-order network can provide auxiliary information for low-order networks but cannot improve classification accuracy.

## 1. Introduction

**FIGURE 1:**
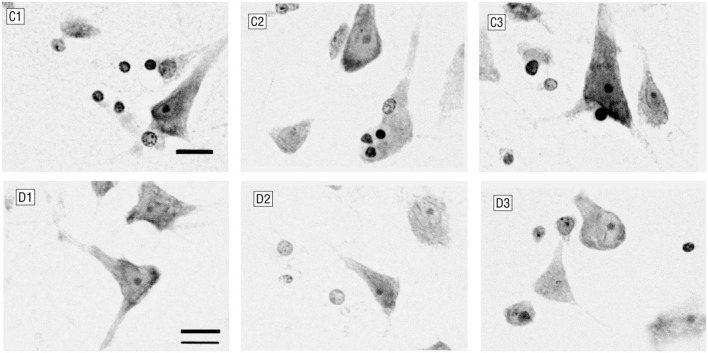
Glial cells and neurons in layer 6 of the anterior cingulate cortex. C1–C3 were control subjects (male, 44 years old), and D1–D3 were patients with major depression (female, 52 years old). Patients with major depression had fewer glial cells and smaller neurons (Nissl staining; bar, 12 μm).

In recent years, because neuroimaging can directly provide *in vivo* brain function and structure information, more and more people have begun to use machine learning technology to extract imaging markers for intelligent diagnosis of major depression disease (MDD) (Gao et al., 2018). However, most of the studies are data-driven, and neither the data selection nor the interpretation of the results pays attention to the histopathological basis of MDD. Despite years of efforts, the pathological and physiological mechanism of MDD itself is still unclear. Many autopsy studies have shown that the density of global glial cells in emotion-related brain regions is decreased in depressed patients (O’Leary and Mechawar, 2021) [as shown in Figure 1: (Cotter et al., 2001)]. The glial cells provide metabolic and regulatory support to neurons, in which astrocytes are responsible for increasing the number of mature and functional synapses (Pannasch et al., 2011). The neural circuit pathways of the brain depend not only on neurons but also on glial cells that significantly affect structural and functional connections (Fields et al., 2015). The latest findings showed a strong correlation between the brain’s microscopic neural circuits and the macroscopic fMRI-based resting-state functional network (Kahali et al., 2021). Therefore, we have reason to believe that the resting-state functional network carrying pathological features is one of the ideal data for the intelligent diagnosis of MDD.

As we all know, the human brain network is composed of different subnets. The whole brain supports functional separation and integration, presenting the so-called small-world attribute (Bassett and Bullmore, 2006), and different hierarchical subnets complete information collection and processing. The medium and low-level systems are responsible for collecting information, and the high-level systems are accountable for integrating and abstracting information. The human brain can change the collection and synthesis of information by adjusting the mental state. For example, the level of attention can change perception, information collection, and synthesis (Keller et al., 2019). Based on this neuropsychological mechanism, someone has developed a high-order functional connectivity (HOFC) network specially used to provide high-level information in the brain network (Zhang et al., 2016). A study showed that HOFC could improve the differences between groups, better capture individual differences, improve the modularity of the brain network, and provide supplementary information for the traditional low-order functional connectivity (LOFC) network. The results showed that multi-layer features extracted from different levels of networks could more accurately identify mild cognitive impairment (Zhang et al., 2016), even early mild cognitive impairment (Zhang et al., 2017). Therefore, we would like to know whether combining the characteristics of different brain networks can provide more abundant information and higher accuracy for the intelligent diagnosis of MDD.

**FIGURE 2:**
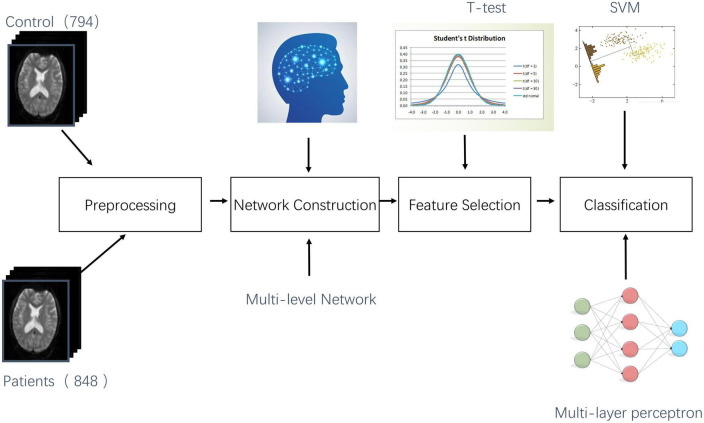
Flow chart of the study.

We use a multicenter, extensive sample data to test our hypothesis in this study. First, we constructed three types of networks: 1. LOFC; 2. topographical information-based high-order functional connectivity [tHOFC (Zhang et al., 2016)] 3. associated HOFC [aHOFC (Zhang et al., 2017)]. Then two samples *t*-test is used to extract the features, and the multi-layer features are fused. Finally, MDD is classified by using a multi-layer perceptron (MLP) or support vector machine (SVM) training classifier. The whole experimental flow is shown in Figure 2.

## 2. Materials and methods

### 2.1. Subjects

All data in this study are from the REST-meta-MDD project. The brain imaging data of 1,300 depressed patients and 1,128 healthy controls through 25 research groups in 17 hospitals in China were collected in this project (Yan et al., 2019). We further screened the data to meet the needs of this study. For detailed methods, please refer to our previous paper (Long et al., 2022). In simple terms, it is to remove data whose repetition time is not 2.0; Delete the data of subjects with time series of 0; Finally, each site’s data is tested for gender and age matching, and the unmatched sites are deleted. This means that the data of all sites in this study have passed the age and gender matching test. In the end, data from 10 sites (1,160 subjects) were included in this study. Table 1 shows the subject information. For more details, please refer to Yan et al. (2019).

**TABLE 1:** Subject information.

Number	1,160	Number of sites	10
Male	434	Female	726
MDD	597	Normal controls	563

Three types of networks are constructed in this study: 1. LOFC, 2. tHOFC, 3. aHOFC. To make the results more universal, we chose the most widely used anatomical automatic labeling (AAL) (Tzourio-Mazoyer et al., 2002) as a brain atlas.

### 2.2. Data pre-processing

The data preprocessing was performed by DPARSF. Global signal regression was performed on all data. The time series were extracted according to the AAL brain atlas, and then the average time series of each brain region was calculated. Please refer to the literature for the detailed data preprocess (Yan et al., 2019).

### 2.3. Definition of brain network

All networks in this study were generated by the BrainNetClass Toolkit (Zhou et al., 2020). To distinguish HOFC, we call Pearson-based functional connectivity (FC) as LOFC, defined as follows: the brain is divided into n regions of interest (ROI) according to the brain atlas. The ith ROI can be expressed as a vector *x*_*i*_ = [*x*_1*i*_,*x*_2*i*_,…,*x*_*Ti*_]′ ∈ *R^T^*(’indicates transposition), the whole brain signal can be expressed by matrix *X* = [*x*_1_,*x*_2_,…,*x*_*N*_] ∈ *R^T^*^×*N*^. The network was expressed as a weighted graph *W* ∈ *R^N^*^×*N*^. Each element in the matrix is a Pearson correlation (PC) between two brain regions. The PC-derived function network is usually used as a benchmark for comparison with other advanced methods. The formula is as follows:


(1) $$F\,C_{i\,j}=\frac{\sum_{t=1}^{T}\left(x_{i}\,\left(t\right)-\bar{x_{i}}\right)\,\left(x_{j}\,\left(t\right)-\bar{x_{j}}\right)}{\sqrt{\sum_{t=1}^{T}{\left(x_{i}\,\left(t\right)-\bar{x_{i}}\right)}^{2}}\,\sqrt{\sum_{t=1}^{T}{\left(x_{j}\,\left(t\right)-\bar{x_{j}}\right)}^{2}}}$$

tHOFC takes the FC between each ROI and all other ROIs as the first-order feature and then calculates the PC between them based on the first-order feature. The obtained coefficient is the HOFC based on the connection topology attribute. The formula is as follows:


(2) $$t\,H\,O\,F\,C_{i\,j}=\frac{\sum_{k}\left(w_{i\,k}-\bar{w_{i.}}\right)\,\left(w_{j\,k}-\bar{w_{j.}}\right)}{\sqrt{\sum_{k}{\left(w_{i\,k}-\bar{w_{i.}}\right)}^{2}}\,\sqrt{\sum_{k}{\left(w_{j\,k}-\bar{w_{j.}}\right)}^{2}}}$$

Among *w*_*i*._ = {*w*_*ik*_|*k* ∈ *N*,*k*≠*i*},*i*,*j*,*k* = 1,2,…,*N*,*k*≠*i*,*j*. Since LOFC is used as the first-order feature instead of the bold time-series signal in the tHOFC calculation, the result is essentially different from that of LOFC. Studies have shown that tHOFC can provide supplementary information for conventional LOFC and help reveal the differences between subjects with mild cognitive impairment (MCI) and normal controls (Zhang et al., 2016, 2019).

Associated high-order functional connectivity is defined based on the mutual relationship between the topological attributes of tHOFC and LOFC, and the calculation method is similar to FC and tHOFC (Eq. 3). It measures the functional correlation between layers (the lower layer and the higher layer). It is a supplement to the information contained in LOFC and tHOFC. Some studies showed that combining these three networks can further improve the diagnostic accuracy of MCI (Zhang et al., 2017). Theoretically, unlike LOFC and tHOFC, the aHOFC matrix is not symmetric, and the self-connection is not 1. However, we find that the upper and lower triangles are highly related. Therefore, to simplify the calculation, we change the aHOFC into a symmetric matrix by W ← (W + W^∧^’) / 2 (Zhou et al., 2020).


(3) $$a\,H\,O\,F\,C_{i\,j}=\frac{\sum_{k}\left(t\,H\,O\,F\,C_{i\,k}-\bar{t\,H\,O\,F\,C_{i.}}\right)\,\left(w_{j\,k}-\bar{w_{j.}}\right)}{\sqrt{\sum_{k}{\left(t\,H\,O\,F\,C_{i\,k}-\bar{t\,H\,O\,F\,C_{i.}}\right)}^{2}}\,\sqrt{\sum_{k}{\left(w_{j\,k}-\bar{w_{j.}}\right)}^{2}}}$$

### 2.4. Feature selection

**FIGURE 3:**
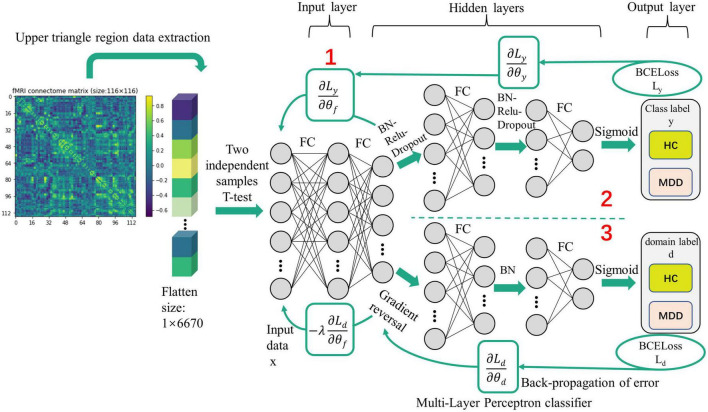
The domain-adversarial training of neural networks.

As shown in the upper left corner of Figure 3, the brain network constructed based on fMRI is a symmetric matrix (size:116 × 116). The upper triangle part is compressed into a one-dimensional vector to form initial features (1 × 6670). Too many indistinguishable features will adversely affect the classification results and reduce the robustness of the model. This study used a two-sample *t*-test to choose the features with discrimination. For learning, the reduced dimension features are sent to the classifier. For the multi-network joint classification, different features produced from three networks are connected to form a vector, the multi-layer feature (Long et al., 2012), and then put into the classifier for training. To ensure the model’s generalization performance, we adopt leave-one-site cross-validation (LOSCV).

### 2.5. Classifier

This study uses two types of mathematical models to construct classifiers: deep learning and support vector machine. The first is the linear support vector machine (SVM). SVM is the most commonly used classification method and has achieved good results on small data sets (Gao et al., 2018). The second is deep learning models. As the classification tasks become more and more complex and the amount of data increases, more complex deep learning models are used for the intelligent diagnosis of MDD. MLP is a typical deep learning model.

The MLP classifier is based on domain-adversarial training of neural networks (DANN). The selected features are sent to the MLP for learning. Changing the size of the convolution weight matrix can achieve the implicit dimensionality reduction of the data in the upper layer, and the generated data is used as the input of the next layer.

The DANN model is implemented based on Pytorch and uses the adam optimizer to train the network model. The learning rate is 0.001. The network is divided into three parts: feature extractor (the first part), label predictor (the second part), and domain classifier (the third part). The details are shown in Figure 3. They use the adversarial relationship between the feature extractor and the domain classifier to mix source and target domain samples in a specific space. After the feature extractor, domain classifier, and label predictor are all trained, the source domain and target domain can be mixed and classified.

The second and third sub-networks are feedforward networks with the same structure. They contain two fully connected convolutional layers and transfer or update feature information through batch normalization (BN), rectification linear unit (ReLU), and Dropout (BN-ReLU-Dropout = 0.5). In parameter information transmission, the number of hidden layer nodes in each layer is 0.5 times the number of hidden layer nodes in the previous layer. Finally, the classification result is obtained through the sigmoid function. The lower part has a particular process called a gradient reversal layer (GRL) which multiplies the error transmitted to this layer by a negative number -λ so that the training objectives of the network before and after GRL will be opposite to achieve the effect of confrontation. The error of the whole network is generated by supervised source domain learning error (L_*y*_) and unsupervised target domain learning error (L_*d*_), both of which are calculated by binary cross entropy loss (BCE_Loss_). Weighted BCE_loss_ solves the class imbalance problem with the super parameter W_*c*_, where c is the class index, defined as (Eqs 4–6):


(4) L=1Na⁢l⁢l∑n=1Na⁢l⁢l(∑c=1cWcE(yn,c,y′n,c)

(5) $$W_{c}=\frac{e^{1/N\,c}}{\sum_{c=1}^{C}e^{1/N\,c}}$$

(6) $$E\,\left(y_{n,c},{\hat{y}}_{n,c}\right)=-\left(y_{n,c}\,l\,o\,g\,{\hat{y}}_{n,c}\right)+\left(1-y_{n,c}\right)\,\log\left(1-{\hat{y}}_{n,c}\right)$$

where L is the weighted BCE_Loss_, N_*c*_ is the sample numbers of class c, N_sall_, and C are the total numbers of samples and classes, and E(Y_nc_,Ŷnc) represents the BCE_Loss_ for the label truth Y_nc_ and the predicted probability Ŷnc.

The random gradient descent optimizer uses the loss gradient calculated by backpropagation to update the network parameters. After many experiments, the GRL is placed between the feature extraction network and the domain classification network. The error transmitted to this layer is multiplied by a negative number- λ. The network training objectives before and after GRL are opposite to achieve the effect of confrontation.

## 3. Results

**FIGURE 4:**
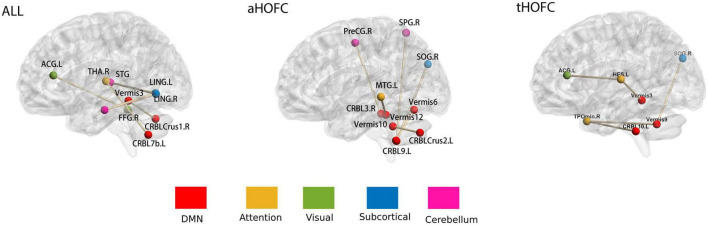
Features selected for networks. The whole brain was divided into six subnets marked with different colors: (1) DMN; (2) attention network; (3) visual network; (4) subcortical network; (5) cerebellum. The brain networks were visualized with the BrainNet Viewer (Xia et al., 2013); see Table 2 for abbreviations of brain regions on the map.

Although the three networks represent different meanings, a single feature in each network is the FC between two brain regions (HOFC or LOFC). There are seven features selected in each round of the three networks (*p* < 0.01). In the aHOFC classification, 26 features were selected in each round, six of which were not seen in other networks and were all related to the cerebellum. In the tHOFC classification, 36 features were selected in each round, five unique, with the highest number of FC between the cerebellum and the temporal lobe. In general, the cerebellum appears most frequently, which indicates that the cerebellum plays a crucial role in the pathological changes of MDD. The changes in the cerebellum and default mode network (DMN), occipital lobe, and frontal lobe can also distinguish MDD from ordinary people. See Figure 4 and Table 2 for more details.

**TABLE 2:** Features selected for networks.

Network	Functional connectivity
ALL	(ACG.L, FFG.R) (LING.L, STG.L)(LING.R, STG.L) (LING.L, STG.R) (THA.R, CRBL7b.L) (CRBLCrus1.R, Vermis3)
aHOFC	(PreCG.R, CRBL3.R) (SPG.R, CRBL9.R) (SOG.R, CRBL9.R) (MTG.L, Vermis12) (CRBL9.L, Vermis6) (CRBLCrus2.L, Vermis10)
tHOFC	(ACG.L, HES.L) (TPOmin.R, CRBL10.L) (HES.L, Vermis3) (SOG.R, Vermis9) (TPOmin.R, Vermis9)

R, right; L, left; ACG, anterior cingulate and paracingulate gyrus; FFG, fusiform gyrus; LING, lingual gyrus; STG, superior temporal gyrus; THA, thalamus; CRBLCrus1, cerebellum crus 1; PreCG, precentral gyrus; CRBL3, cerebellum superior 3; SPG, superior parietal gyrus; CRBL9, cerebellum inferior 9; SOG, superior occipital gyrus; MTG.L, middle temporal gyrus; CRBLCrus2, cerebellum crus 2;HES, Heschl gyrus; TPOmin, temporal pole, middle temporal gyrus; CRBL10, cerebellum inferior 10. ALL shows the features selected in each round of the three networks; aHOFC showed the unique features chosen in each round of the aHOFC; tHOFC showed the unique features selected in each round of the tHOFC.

**FIGURE 5:**
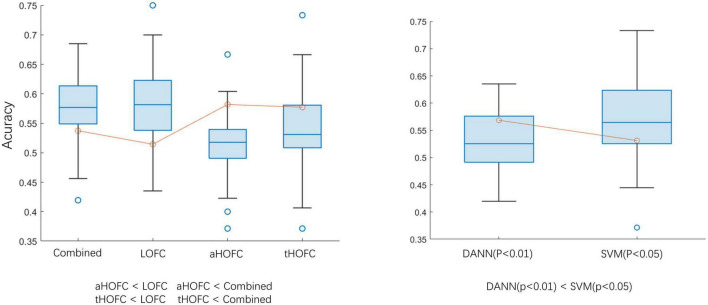
Classification accuracy comparison. aHOFC: all classification results based on aHOFC; tHOFC: all classification results based on tHOFC; LOFC: all classification results based on LOFC; Combined: classification result of three network features fusion. DANN (*p* < 0.01): using DANN as a classifier, the *p*-value of feature selection is 0.01; SVM (*p* < 0.05): using SVM as the classifier, the *p*-value of feature selection is 0.05. The inequalities represent statistical differences between the two groups of data.

In this experiment, two *p*-values were selected for feature selection (*p* < 0.01 and *p* < 0.05). When *p* < 0.05, using SVM as a classifier can achieve a classification accuracy of 60.25. The classification ability of different levels of networks is LOFC > HOFC (*P* < 0.05). The test results in Figure 5 show no statistical difference between the classification efficiency of LOFC and the combined networks (*P* > 0.05), indicating that the other two networks do not increase the classification ability. The classification results are similar based on aHOFC and tHOFC networks, but both are worse than multi-layer network classification. The choice of classifier (SVM or DANN) does no effect accuracy. During feature selection, *p*-value selection (0.01 or 0.05) has no effect on classification results. When the classifier is SVM, and feature selection is *p* < 0.05, the classification effect is better than that of DANN (*p* < 0.01). Epoch’s best results are generally higher than the best test results, which suggests that we still need to find the best time to stop searching for the optimal solution during training. See Figure 5 and Table 3 for detailed results.

**TABLE 3:** Average Classification accuracy.

	DANN (test)		SVM		DANN (best_test)	
	*p* < 0.01	*p* < 0.05	*p* < 0.01	*p* < 0.05	*p* < 0.01	*p* < 0.05
tHOFC	52.22 ± 3.77	53.64 ± 8.59	53.64 ± 4.47	55.44 ± 9.49	60.49 ± 6.91	60.12 ± 6.79
aHOFC	50.59 ± 4.68	52.89 ± 6.75	49.94 ± 5.09	52.33 ± 6.82	57.91 ± 5.77	60.53 ± 5.24
LOFC	55.66 ± 5.89	57.83 ± 5.28	59.00 ± 7.26	60.25 ± 5.86	63.86 ± 6.84	62.49 ± 7.74
Combined	54.02 ± 7.29	58.36 ± 3.27	59.14 ± 3.07	59.38 ± 5.28	60.62 ± 7.50	61.93 ± 7.95

DANN (test): classification result of domain-adversarial training of neural networks when the training accuracy is the highest. SVM, support vector machine; DANN (best_test), the highest accuracy with each epoch using domain-adversarial training of neural networks; combined, connect the features selected by the three networks and then classify; Unit is the percentage (%).

## 4. Discussion

To the best of our knowledge, this is the first time to use deep learning technology to realize MDD automatic classification in combination with a multi-layer network (Rahaman et al., 2020; Zhang et al., 2021; Chen et al., 2022). The results show that the classification performance of low-order networks is higher than that of high-order networks. The aHOFC and tHOFC can provide new information for LOFC, but integration cannot improve classification performance.

Studies have shown that tHOFC (Zhang et al., 2016) and aHOFC (Zhang et al., 2017) are beneficial supplements to LOFC. This study proves this again. For example, we found that the cerebellar-cingulate gyrus is the most discriminative feature in the aHOFC network, which may reflect the disorder of the cerebellar-cortical-limbic circuit in MDD patients, leading to emotional and cognitive impairment. This result is consistent with previous studies (Lai and Wu, 2014). However, this FC does not appear in the LOFC network, which indicates that HOFC can provide other important information for LOFC.

We combine multiple networks at different levels for classification, but the classification ability of integrated features is similar to that of individual parts. It is possible that the simplicity of the feature fusion method is the cause of this issue. Future research needs to design more sophisticated ways to fuse features, stimulating the advantages of multi-level network features and improving classification performance. Previous studies have found that the classification efficiency of HOFC is higher than that of LOFC (Yan et al., 2019). However, these results did not appear in this study. The following reasons may cause this: (1) The sample size is different. This experiment is based on multicenter large data samples and is tested separately on an independent test set. Previous studies were based on small samples; and (2) Different disease types. This study is to classify MDD, and prior studies have classified Alzheimer’s disease (AD). Although they are both mental diseases and may have some common pathological features, the two conditions differ. The results of AD may not be generalized to MDD. It also suggests that future research should develop more robust and generalized network models to classify neuropsychiatric diseases.

The classification accuracy of this study is low, and most of the classification accuracy is below 60%. There are two main reasons for this situation: (1) To ensure our conclusions’ robustness and the classifier’s generalization, we use big data from 10 sites. Different machine models, scanning parameters, and equipment status will reduce the accuracy of multi-site data classification. Therefore, the low accuracy is also reasonable. (2) Our cross-validation method adopts LOSCV. We train the model by extracting data from other research groups and classifying brand-new data. Although this can ensure the classifier ‘s generalization, it reduces the accuracy of the results.

Another significant contribution of this study was selecting FCs that genuinely represent the pathological changes of MDD. Our results indicated that the FC changes between the cerebellum and occipital lobe were the most distinguishing features. Studies have shown that the MDD group demonstrated decreased cerebellar–cerebral FC with the prefrontal lobe and DMN and increased FC with visual recognition network (lingual gyrus, middle occipital gyrus, and fusiform) (Guo et al., 2013). This enhancement has been typically viewed as either a compensatory reallocation (Cabeza et al., 2002; Grady et al., 2005) or dedifferentiation (Logan et al., 2002) which the increased FC between the cerebellum and the visual recognition network may compensate for the decrease in the cerebellar–cerebral FC (Liu et al., 2012). Our results indicated that the FC alteration was likely to be used to identify MDD.

This study has many limitations. First, we chose MLP as the classifier. Since we use network data, the recently emerged graph neural network is suitable for processing such data. Future research should use more robust models for MDD classification. Secondly, we only used fMRI data in this study. Clinical features and gene information are also crucial for the classification of MDD. Future research should integrate these data into the classification framework to improve accuracy. Thirdly, there is no difference between the classification results of SVM and MLP. This may be because our sample size is not large compared with the database like ImageNet. As the sample size gradually increases, DP will become more and more competent for this classification in the future.

## 5. Conclusion

This study wants to know whether the integration of three different levels of networks can improve the performance of MDD intelligent diagnosis. Experimental results show that combining different layers of networks cannot improve classification accuracy, but higher-order networks can provide new features for classification.

## Data availability statement

The datasets presented in this study can be found in online repositories. The names of the repository/repositories and accession number(s) can be found below: http://rfmri.org/REST-meta-MDD.

## Ethics statement

The studies involving human participants were reviewed and approved by http://rfmri.org/REST-meta-MDD. The patients/participants provided their written informed consent to participate in this study.

## Author contributions

FL completed the theoretical research, technical method design, and manuscript revision. DL and MZ completed the technical method design, experiment and analysis, and the writing and revision of the manuscript. FC, JY, and QZ conducted research guidance, technical method demonstration, and manuscript review. All authors contributed to the article and approved the submitted version.
